# Clotrimazole Targets the c-Myc–Survivin Axis to Reduce the Viability of Ovarian Cancer Stem Cells Alone and in Combination with Chemotherapeutic Agents

**DOI:** 10.3390/ijms27146175

**Published:** 2026-07-10

**Authors:** Yasufumi Ito, Kazuki Nakamura, Yurika Nakagawa-Saito, Shuhei Suzuki, Yuta Mitobe, Senri Takenouchi, Keita Togashi, Asuka Sugai, Manabu Seino, Tsuyoshi Ohta, Satoru Nagase, Chifumi Kitanaka, Masashi Okada

**Affiliations:** 1 Department of Molecular Cancer Science, Yamagata University School of Medicine, 2-2-2 Iida-nishi, Yamagata 990-9585, Japan; 2 Department of Obstetrics and Gynecology, Yamagata University School of Medicine, 2-2-2 Iida-nishi, Yamagata 990-9585, Japan; 3 Department of Neurosurgery, Yamagata University School of Medicine, 2-2-2 Iida-nishi, Yamagata 990-9585, Japan; 4 Department of Clinical Oncology, Yamagata Prefectural Shinjo Hospital, 720-1 Kanazawa, Shinjo 996-8585, Japan; 5 Department of Ophthalmology and Visual Sciences, Yamagata University School of Medicine, 2-2-2 Iida-nishi, Yamagata 990-9585, Japan; 6 Institute for Promotion of Medical Science Research, Yamagata University Faculty of Medicine, 2-2-2 Iida-nishi, Yamagata 990-9585, Japan

**Keywords:** epithelial ovarian cancer, baculoviral inhibitor of apoptosis repeat-containing 5, azole, endometrioid, clear cell

## Abstract

Ovarian cancer stem cells (OvCSCs) are one of the main factors contributing to post-treatment recurrence and the poor prognosis of patients with ovarian cancer. Therefore, the development of therapeutic strategies targeting OvCSCs is needed to improve patient survival. We previously reported the high expression of survivin/*BIRC5* in OvCSCs and also that targeting pathways regulating survivin expression effectively suppressed OvCSC survival. In the present study, we tested a panel of agents consisting of FDA-approved drugs and compounds under clinical studies for their ability to suppress survivin expression in OvCSCs and identified clotrimazole (CTZ) as a potent candidate. The effects of CTZ on survivin expression were examined by RT-PCR and Western blot analyses. The effects of CTZ alone or in combination with anticancer agents on OvCSCs were evaluated using WST-8, PI uptake, and colony formation assays. CTZ preferentially impaired OvCSC survival by suppressing the c-myc–survivin axis without affecting normal fibroblasts and enhanced the efficacy of platinum- and taxane-based chemotherapeutic agents. These results suggest that CTZ suppresses survivin expression in OvCSCs and enhances the effects of conventional ovarian cancer chemotherapeutic agents, supporting further investigations of this approach as a potential strategy for ovarian cancer treatment.

## 1. Introduction

Ovarian cancer is the most lethal gynecologic malignancy and the eighth most common cancer among women, with a 5-year survival rate as low as 10–40% [1]. Despite significant advances in treatment, the recurrence rate of ovarian cancer remains high, with more than 80% of advanced-stage cases recurring within 2 years [2]. Ovarian cancer stem cells (OvCSCs) are cancer stem cells (CSCs) in ovarian cancer and represent a rare subpopulation of tumor cells that exhibit higher resistance to chemotherapy and contribute more strongly to tumor recurrence than non-cancer stem cells (non-CSCs). OvCSCs have emerged as one of the key drivers of ovarian cancer recurrence; therefore, their elimination represents a promising approach for improving the survival outcomes of patients with ovarian cancer [3,4,5].

The expression of survivin/*BIRC5*, a member of the IAP family, is low in normal adult tissues, but is high in the majority of human malignancies, where it functions to inhibit apoptosis and promote cell proliferation; therefore, survivin has long been regarded as an attractive therapeutic target and has been extensively investigated over the years [6,7,8]. We previously reported the high expression of survivin in OvCSCs and also that its suppression induced cell death [9]. Using drug repositioning techniques, we identified compounds that target survivin in OvCSCs and showed that these compounds enhanced the cytotoxic effects of paclitaxel (PTX) and carboplatin (CBDCA), the standard treatments for ovarian cancer [9,10].

These findings highlight the therapeutic potential of targeting survivin in OvCSCs, and indicate that the identification of clinically applicable survivin-targeting agents may provide a meaningful strategy to improve treatment outcomes for ovarian cancer. In the present study, we tested a panel of agents consisting of FDA-approved drugs and compounds under clinical studies for their ability to suppress survivin expression in OvCSCs, and then examined the effects of the identified drug and its combination with taxane- and platinum-based agents.

## 2. Results

### 2.1. Clotrimazole (CTZ) Reduces Survivin Expression in OvCSCs

**Figure 1:**
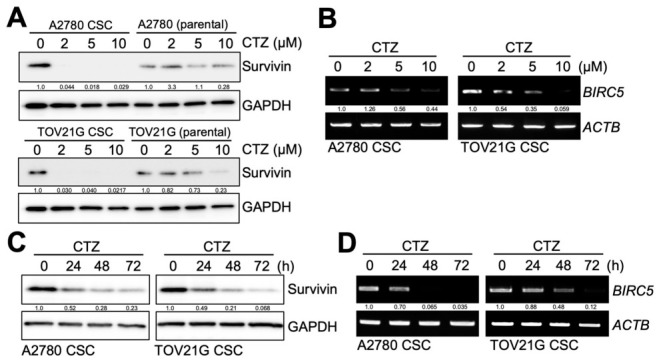
Clotrimazole (CTZ) reduces survivin expression in dose- and time-dependent manners in ovarian cancer stem cells (OvCSCs). (**A**) OvCSCs (A2780 CSC and TOV21G CSC) and their parental cells (A2780 and TOV21G) treated with the indicated concentrations of CTZ for 72 h (h) were analyzed by Western blotting for survivin and GAPDH. (**B**) OvCSCs treated with the indicated concentrations of CTZ for 48 h were subjected to an RT-PCR analysis for *BIRC5* (encoding survivin) and *ACTB* (encoding β-actin). OvCSCs treated with 5 μM CTZ for 24–72 h were subjected to Western blot (**C**) or RT-PCR (**D**) analyses for the indicated proteins or mRNAs. The numbers below Western blot and RT-PCR images represent relative band intensities after each band was quantified by densitometry and normalized to the GAPDH (Western blot) or *ACTB* (RT-PCR) value. Representative results from two independent experiments are shown.

To identify clinically translatable candidates for drug repositioning, we initially evaluated a panel consisting of FDA-approved drugs, compounds under clinical investigation, and clinically relevant analogs for their ability to reduce the expression of survivin, which is high in OvCSCs and contributes to their survival. We found that CTZ, an FDA-approved broad-spectrum antifungal agent [11,12], consistently reduced the expression of survivin in both A2780 CSC and TOV21G CSC, which are OvCSC cell lines. We examined the range of CTZ concentrations that were not toxic to IMR90 cells, a well-established non-transformed human cell line used as a control for toxicity assessments. CTZ did not significantly reduce IMR90 cell viability at concentrations up to 20 μM (Figure 3A). Clinical studies previously reported that the maximum plasma concentration of CTZ that did not cause serious adverse events was approximately 2–5 μM [13,14]. Therefore, subsequent experiments were conducted using clinically relevant concentrations, while 20 μM was included only in initial dose–response cell viability assays. Using A2780 and TOV21G CSCs as well as their parental cells, which are predominantly non-CSCs, we found that CTZ dose-dependently decreased survivin protein expression levels in both CSCs (Figure 1A). In contrast, under these conditions, the treatment of parental cells with CTZ did not markedly affect survivin expression or only resulted in a modest reduction (Figure 1A). To establish whether the CTZ-induced reduction in survivin in OvCSCs was caused by changes at the transcriptional level, we performed a Reverse Transcription (RT)-PCR analysis. The results obtained showed that survivin/*BIRC5* mRNA levels decreased in a CTZ dose-dependent manner, consistent with the reduction observed at the protein level (Figure 1B). We then investigated the time-dependent effects of CTZ on the reduction in survivin in OvCSCs using Western blotting and RT-PCR analyses. A time-course analysis revealed that the decrease in survivin/*BIRC5* mRNA levels occurred simultaneously with or prior to the reduction in survivin protein levels (Figure 1C,D). Collectively, these results demonstrate that CTZ reduced survivin/*BIRC5* expression in OvCSCs at concentrations that were not toxic to IMR90 cells.

### 2.2. CTZ Suppresses the c-Myc–Survivin Axis in OvCSCs

**Figure 2:**
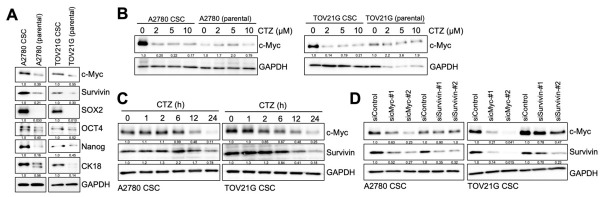
Clotrimazole (CTZ) suppresses the c-myc–survivin axis in ovarian cancer stem cells (OvCSCs). (**A**) OvCSCs (A2780 CSC and TOV21G CSC) and their parental cells (A2780 and TOV21G) were subjected to a Western blot analysis for the indicated proteins. (**B**) Cells treated with the indicated concentrations of CTZ for 72 h (h) were subjected to a Western blot analysis for c-Myc and GAPDH. (**C**) Cells treated with 10 μM CTZ for the indicated times were subjected to a Western blot analysis for the indicated proteins. (**D**) Cells transfected with siRNA against c-Myc (120–160 pmol per 6 cm dish) or survivin (80–120 pmol per 6 cm dish) or with control siRNA (siControl: 160 pmol per 6 cm dish) were subjected to a Western blot analysis for the indicated proteins. The numbers below Western blot images represent relative band intensities after each band was quantified by densitometry and normalized to the GAPDH value. Representative results from two independent experiments are shown.

We next investigated the molecular mechanisms underlying the CTZ-induced reduction in survivin in OvCSCs. Based on previous findings showing that CTZ suppressed c-myc expression [15,16] and that c-myc positively regulated the transcription of survivin [17,18], we hypothesized that the CTZ-induced suppression of c-myc may reduce survivin expression in OvCSCs. To test this hypothesis, we first compared c-myc expression levels in OvCSCs and their parental cells. The expression levels of the representative CSC-associated markers SOX2, OCT4, Nanog, and CK18, a marker reported to be specifically expressed in OvCSCs [19,20], were higher in A2780 CSCs and TOV21G CSCs than in their parental cells (Figure 2A). In addition, consistent with our previous findings, survivin was highly expressed in OvCSCs [9], and c-myc expression correlated with that of survivin (Figure 2A). Based on these results, we examined c-myc expression following the CTZ treatment and found that, similar to the expression pattern of survivin, CTZ markedly decreased c-myc protein levels in OvCSCs in a dose-dependent manner, whereas c-myc expression in their parental cells was largely unaffected or only slightly reduced (Figure 2B). These results support the hypothesis that the CTZ-induced reduction in survivin is mediated through the suppression of c-myc, which is known to function as an upstream regulator of survivin and is highly expressed in OvCSCs. We also performed a time-course analysis following the CTZ treatment and found that c-myc expression was reduced prior to that of survivin (Figure 2C). Furthermore, knockdown experiments for each gene showed that the knockdown of c-myc reduced survivin expression, whereas that of survivin only resulted in a limited or minimal reduction in c-myc expression, indicating that c-myc acted upstream of survivin to positively regulate its expression in the OvCSCs used in this study (Figure 2D). Taken together, these results suggest that the CTZ treatment suppressed the c-myc–survivin axis in OvCSCs.

### 2.3. CTZ Suppresses OvCSC Survival by Down-Regulating Survivin Expression

**Figure 3:**
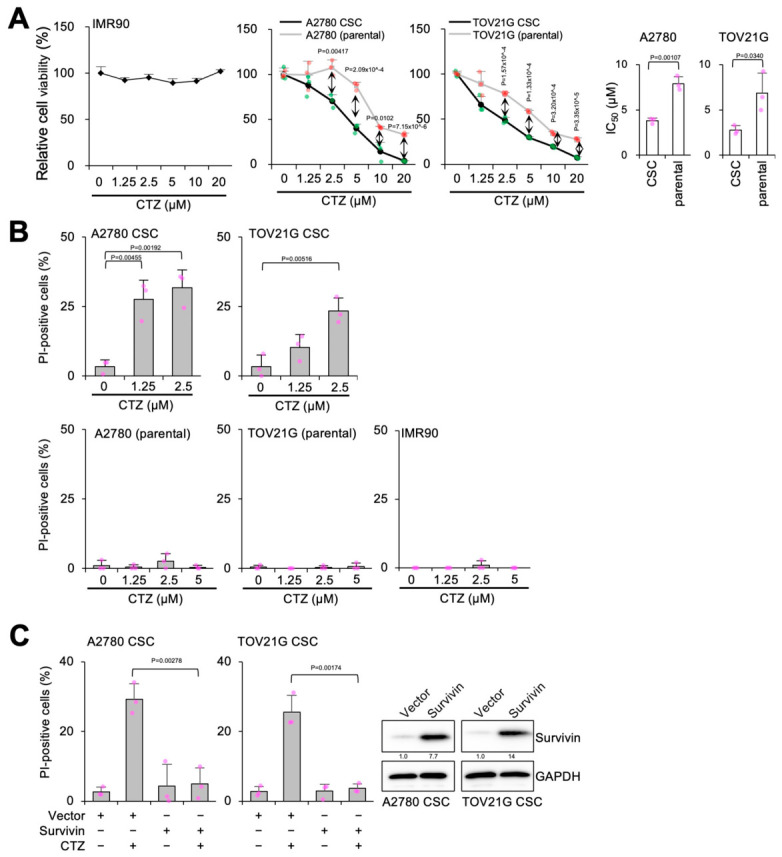
Clotrimazole (CTZ) reduces viability and induces death in ovarian cancer stem cells (OvCSCs). (**A**) IMR90 cells and OvCSCs (A2780 CSC and TOV21G CSC) and their parental cells (A2780 and TOV21G) treated with CTZ at the indicated concentrations for 72 h were subjected to a WST-8 assay (Left graphs). Exact *p* values are shown for each comparison (indicated with a double-headed arrow) between OvCSCs and their parental cells treated with CTZ at the same concentrations. Bars represent the mean IC_50_ + SD of 3 independent experiments, calculated by the linear interpolation method (Right graphs). (**B**) Cells treated with CTZ as in (**A**) were subjected to a propidium iodide (PI) uptake assay. (**C**) Cells were transfected with pEGFP-C1 (Vector) or pIRES2- Survivin/EGFP (Survivin). One day later, cells were treated with 5 μM CTZ for 48 h and then subjected to flow cytometry to assess the percentage of PI-positive cells (Left graphs) or to a Western blot analysis for survivin and GAPDH (Right panels). *p* values are indicated in the figures and were assessed by the two-tailed *t*-test. The numbers below Western blot images represent relative band intensities after each band was quantified by densitometry and normalized to the GAPDH value. n = 3 biological replicates per group. Representative results from two independent experiments are shown.

We then investigated the effects of CTZ on the viability of OvCSCs and their parental cells. To achieve this, we examined the viability of cells treated with CTZ using a WST-8 assay. As described above, CTZ did not affect the viability of IMR90 cells at concentrations up to 20 μM (Figure 3A); however, it suppressed the viability of OvCSCs and their parental cells in a dose-dependent manner (Figure 3A). OvCSCs, which expressed higher levels of survivin than their parental cells, were also more sensitive to the CTZ treatment (IC_50_: A2780 CSC, 3.8 μM; A2780, 7.9 μM; TOV21G CSC, 2.7 μM; TOV21G, 6.9 μM) (Figure 3A). Using the same treatment conditions, PI uptake assays showed that CTZ dose-dependently induced cell death in OvCSCs without affecting IMR90 cells (Figure 3B), whereas little or no cell death was observed in their parental cells (Figure 3B). To establish whether the reduction in survivin contributed to CTZ-induced cell death in OvCSCs, we investigated whether survivin overexpression attenuated CTZ-induced cell death. As expected, survivin overexpression suppressed CTZ-induced cell death in OvCSCs (Figure 3C). These results demonstrate that the reduction in survivin contributed to CTZ-induced cell death in OvCSCs. Collectively, these results suggest that CTZ suppressed OvCSC survival and induced cell death by suppressing the expression of survivin.

### 2.4. CTZ Enhances Sensitivity to CBDCA and PTX

**Figure 4:**
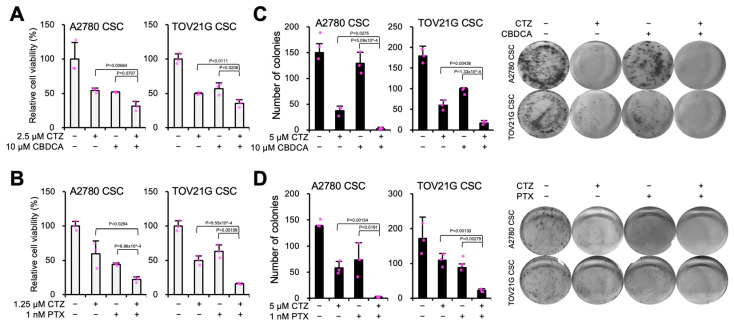
Clotrimazole (CTZ) sensitizes ovarian cancer stem cells (OvCSCs) to carboplatin (CBDCA) and paclitaxel (PTX). OvCSCs (A2780 CSC and TOV21G CSC) treated with CTZ and CBDCA (**A**) or PTX (**B**) for 72 h were subjected to a WST-8 assay. Cells were treated as in (**A**,**B**). After being cultured for 72 h, cells were cultured for 144 h in the absence of drugs for the colony formation assay (**C**,**D**). *p* values are indicated in the figures and were assessed by the two-tailed *t*-test. n = 3 biological replicates per group. Representative results from two independent experiments are shown.

We previously reported that targeting survivin in OvCSCs enhanced sensitivity to taxanes and platinum agents, which are used as standard therapies for ovarian cancer [9,10]. Therefore, we performed combination experiments to investigate whether CTZ, an FDA-approved drug that we identified in this study as exerting suppressive effects on the c-myc–survivin axis, enhanced the efficacy of PTX and/or CBDCA, conventional ovarian cancer chemotherapeutic agents. A treatment with CBDCA, PTX, or CTZ alone reduced the viability of OvCSCs, whereas combination treatment with CTZ decreased cell viability significantly more than each single treatment (Figure 4A,B). Similarly, colony formation assays showed that the combination of CTZ with CBDCA or PTX reduced the clonogenic survival of OvCSCs more than each single treatment (Figure 4C,D). Taken together, these results demonstrate that CTZ, which suppresses the c-myc–survivin axis in OvCSCs, sensitized these cells to taxane- and platinum-based chemotherapeutic agents.

## 3. Discussion

We herein identified CTZ by screening for compounds that inhibit the expression of survivin, an anti-apoptotic factor known to be highly expressed in OvCSCs, and subsequently evaluated its biological effects. We also demonstrated for the first time that CTZ reduced survivin expression by suppressing c-myc and induced cell death in OvCSCs at concentrations that did not affect normal fibroblasts. In addition, we showed that CTZ enhanced the sensitivity of OvCSCs to platinum- and taxane-based therapies, which are used as standard treatments for ovarian cancer.

CTZ is an FDA-approved broad-spectrum antifungal agent that targets ergosterol biosynthesis and thereby disrupts the structure and function of the fungal cell membrane [11,12]. However, its potential as an anticancer agent has also been discussed [21]. For example, CTZ has been reported to induce apoptosis in multiple myeloma cells through increased ROS production, G0/G1 cell-cycle arrest, and the inhibition of NF-κB signaling [22]. Liu et al. demonstrated that CTZ suppressed the invasion and migration of hepatocellular carcinoma cells by inhibiting ERK phosphorylation [23]. In addition, Ochioni et al. emphasized the off-target effects of CTZ and reported that CTZ exerted cytotoxic effects by negatively regulating PI3K signaling, thereby inhibiting glycolysis in melanoma cells, and further suppressed tumor growth in mouse models [24]. Similar to these findings in melanoma, CTZ has been shown to suppress proliferation by inhibiting glycolytic metabolism in breast cancer [25] and glioblastoma [26]. In contrast, the antitumor activity of CTZ in ovarian cancer has not been reported, and, to the best of our knowledge, the effects of CTZ on cancer stem/initiating cells remain unknown. In the present study, using ovarian cancer-derived CSC models, we demonstrated for the first time that CTZ potently suppressed the survival of OvCSCs. Furthermore, we showed that CTZ suppressed c-myc expression in OvCSCs. Since c-myc functions downstream of ERK and PI3K signaling and plays important roles in maintaining CSC survival and glycolytic metabolism [27,28,29], the potent cytotoxic effects of CTZ against OvCSCs appear to be consistent with previous findings. Although studies that investigated the antitumor effects of CTZ generally used concentrations in the several tens of micromolar range [22,23,24,25,26], the IC_50_ values against OvCSCs in the present study were very low, falling within the low micromolar range, which is close to the reported maximum plasma concentrations (2–5 μM) [13,14]. One possibility is that, as demonstrated in the present study, OvCSCs express higher levels of c-myc and survivin, both of which are suppressed by CTZ, than parental cells. Although we found that CTZ suppressed the c-myc–survivin axis in OvCSCs, the mechanisms underlying this effect have yet to be elucidated in detail. Specifically, it remains unclear whether CTZ directly interacts with c-myc, indirectly regulates this pathway through upstream signaling molecules, such as ERK and PI3K, or exerts its effects through other unidentified molecular targets. Given that azole antifungal agents, including CTZ, have been reported to inhibit KCa3.1, a Ca^2+^-gated K^+^ channel [30], the cytotoxic effects of CTZ observed in various tumor cells may, at least in part, be mediated through the inhibition of KCa3.1. However, the anti-asthmatic agent senicapoc, a structurally related KCa3.1 inhibitor, induced little to no consistent reduction in survivin expression in OvCSCs and failed to suppress OvCSC viability at concentrations that did not affect IMR90 cell viability (unpublished data). In addition, Zuccolini et al. reported that the inhibitory effects of CTZ and senicapoc on cancer cell survival and migration were independent of their suppressive effects on plasma membrane-localized KCa3.1 [31]. Collectively, these findings suggest that further studies are required to identify the direct molecular target of CTZ in various tumor cells, including OvCSCs.

Survivin has long been known to contribute to the inhibition of apoptosis and regulation of cell division, and, thus, has been extensively investigated as a therapeutic target in cancer treatment [6,7,8]. In addition, the inhibition of survivin may be effectively combined with conventional cancer therapies [7,8,9,10]. Recent studies from multiple groups, including ours, reported the high expression of survivin in various CSCs, including OvCSCs, and also that the inhibition of survivin expression suppressed CSC survival and stem cell properties [9,32,33,34,35]. Therefore, the development of therapeutic strategies targeting survivin may be highly beneficial for improving the prognosis of not only patients with ovarian cancer, but also those with other malignancies. In the present study, we found that CTZ suppressed survivin expression and inhibited the survival of OvCSCs. Although survivin expression is regulated by multiple upstream signaling pathways [8], c-myc was more highly expressed in the OvCSC cell lines used in this study than in their parental cells, and our experimental results further suggest that c-myc contributes to the regulation of survivin expression in OvCSCs. FGF2/FGFR signaling is widely known to contribute to the stem cell properties of various CSCs [36,37,38], and c-myc has been reported to positively regulate survivin expression downstream of FGF2 signaling [17]. In addition, c-myc has been reported not only to function as a core regulatory factor of stemness programs in cancer cells [39,40], but is also more highly expressed in OvCSCs than in non-OvCSCs [41,42]. Furthermore, there is a growing body of evidence suggesting that the combination of c-myc inhibition with conventional cancer therapies enhances therapeutic efficacy [43,44,45]. Taken together, these findings suggest that targeting the c-myc–survivin axis represents a promising direction for further preclinical investigations on OvCSCs, with CTZ serving as one potential approach to target this pathway.

The systemic administration of CTZ to humans has been reported in the context of sickle cell disease, where it is used to inhibit the K^+^ channel activity of KCa3.1 and thereby prevent K^+^ loss and cellular dehydration in sickled erythrocytes [13,14,46]. Notably, the IC_50_ values of CTZ against OvCSCs identified in the present study (2–4 μM) were close to the reported maximum plasma concentrations (2–5 μM) achieved at doses that did not cause significant adverse effects in humans [13,14]. Although it remains unclear whether KCa3.1 is involved in the regulation of c-myc or survivin expression in OvCSCs, the present results collectively suggest that CTZ-based targeted therapy against OvCSCs is achievable at relatively safe doses associated with minimal adverse effects. In addition, studies aimed at improving the oral bioavailability of CTZ, including β-cyclodextrin and nanoparticle inclusion formulations, are currently being pursued [47]. Since the present study did not include the preclinical experiments necessary to validate the clinical significance of the results obtained, further investigations, including the optimization of dosing schedules and administration methods, as well as the evaluation of potential adverse effects and drug–drug interactions associated with cytochrome P450 inhibition, will be required in the future.

In conclusion, we herein identified CTZ as a therapeutic agent targeting survivin, which we previously identified as one of the vulnerabilities of OvCSCs. Furthermore, the present study demonstrated that CTZ not only impaired OvCSC survival by targeting the c-myc–survivin axis, but also enhanced the efficacy of platinum- and taxane-based standard therapies. Although the effective concentrations of CTZ against OvCSCs were close to previously reported clinically achievable plasma concentrations, further preclinical studies, including in vivo analyses, are required to elucidate the translational potential of CTZ in OvCSCs.

## 4. Materials and Methods

### 4.1. Reagents and Antibodies

CTZ (23593-75-1) was purchased from Tokyo Chemical Industry Co., Ltd. (Tokyo, Japan), CBDCA (S1215) from Selleck Chemicals (Houston, TX, USA), and PTX (P0093) from FUJIFILM Wako Pure Chemical Corporation (Osaka, Japan). CTZ, CBDCA, and PTX were dissolved in DMSO to prepare 10, 10, and 1 mM stock solutions, respectively. An antibody against CK18 (NBP1-47817) was purchased from Bio-Techne (Minneapolis, MN, USA). Antibodies against c-Myc (#13987), GAPDH (#5174), Nanog (#4903), OCT4 (#2890), SOX2 (#3579), and Survivin (#2808) were obtained from Cell Signaling Technology, Inc. (Beverly, MA, USA). Horseradish peroxidase (HRP)-conjugated rabbit and mouse secondary antibodies were from Jackson ImmunoResearch, Inc. (West Grove, PA, USA).

### 4.2. Cell Culture

The establishment and characterization of the human OvCSCs used in this study (A2780 CSCs and TOV21G CSCs) have been described elsewhere [48,49]. OvCSCs were maintained under previously reported monolayer stem cell culture conditions [48,49]. The parental cell lines, A2780 and TOV21G were maintained as previously described [10,50]. IMR90, a human normal fetal lung fibroblast cell line, was obtained from the American Type Culture Collection (Manassas, VA, USA) and maintained in DMEM supplemented with 10% FBS (Thermo Fisher Scientific, Waltham, MA, USA). All IMR90 experiments were performed using cells with a low passage number (<8).

### 4.3. Western Blot Analysis

A Western blot analysis was conducted as previously described [51,52]. Cells were harvested, washed with ice-cold PBS, and then lysed in RIPA buffer (10 mM Tris/HCl (pH 7.4), 0.1% sodium deoxycholate, 0.1% sodium dodecyl sulfate (SDS), 1% Nonidet P-40, 150 mM NaCl, 1 mM EDTA, 10 mM sodium fluoride, 1.5 mM sodium orthovanadate, 10 mM sodium pyrophosphate, and protease inhibitor cocktail set III (FUJIFILM Wako Chemicals, Osaka, Japan)). The lysate was immediately mixed with the same volume of 2 × Laemmli buffer (125 mM Tris/HCl (pH 6.8), 4% SDS, and 10% glycerol) and incubated at 95 °C for 10 min. Samples with protein concentrations measured using a BCA protein assay kit (Thermo Fisher Scientific) were loaded at 10 μg protein per lane, separated by SDS/polyacrylamide gel electrophoresis, and transferred to polyvinylidene difluoride membranes (Immobilon-P, Merck KGaA, Darmstadt, Germany). The membranes were reacted with primary antibodies followed by appropriate HRP-conjugated secondary antibodies, and then detected using Immobilon Western Chemiluminescent HRP Substrate (Merck). Regarding the reprobing of immunoblots, antibodies were stripped from the probed membrane using stripping buffer (2% SDS, 100 mM β-mercaptoethanol, and 62.5 mM Tris-HCl (pH 6.8)). After stripping, the membranes were washed with Tris-buffered saline with Tween 20, blocked with skim milk, and reprobed with appropriate antibodies. Immunoreactive bands were detected using a ChemiDoc Touch device (Bio-Rad Laboratories, Inc., Hercules, CA, USA). Quantification of the bands on the membranes was performed by densitometry using ImageJ software (version 1.53a) (https://imagej.net/ij/, accessed on 6 July 2026).

### 4.4. An RT-PCR Analysis

An RT-PCR analysis was conducted as previously described [52]. Total RNA was extracted from cells using Trizol (Thermo Fisher Scientific Inc.), and 1 μg of total RNA was reverse-transcribed using the PrimeScript RT reagent kit (Takara Bio Inc., Shiga, Japan) according to the manufacturer’s protocol. The target genes were amplified with Quick Taq HS DyeMix (Toyobo Co., Ltd., Osaka, Japan) using the following primers: *BIRC5*, 5′-CCTTTCTCAAGGACCACCGCATC-3′ (forward) and 5′-CGTCATCTGGCTCCCAGCCTT-3′ (reverse); *ACTB*, 5′-CCCATGCCATCCTGCGTCTG-3′ (forward) and 5′-CGTCATACTCCTGCTTGCTG-3′ (reverse).

### 4.5. Transfection of siRNA or Plasmids

AllStars Negative Control siRNA (QIAGEN, Venlo, The Netherlands) or siRNAs against survivin (*BIRC5*: #1 HSS179403, #2 HSS179404) and c-Myc (*MYC*: #1 HSS106837, #2 HSS106839) (Thermo Fisher Scientific) were transfected using Lipofectamine RNAiMAX (Thermo Fisher Scientific) in accordance with the manufacturer’s instructions. siRNAs against survivin (*BIRC5*; 80–120 pmol per 6 cm dish), c-myc (*MYC*: 120–160 pmol per 6 cm dish), or Control (siControl: 160 pmol per 6 cm dish) were transfected. The plasmid pIRES2-Survivin/EGFP (4 μg per 6 cm dish) [53] and the control plasmid pEGFP-C1 (2 μg per 6 cm dish) (GenBank accession #U55763; Takara Bio Inc., Shiga, Japan) were transiently transfected using Lipofectamine 2000 (Thermo Fisher Scientific) in accordance with the manufacturer’s instructions.

### 4.6. Cell Viability/Death Assays

We conducted the WST-8 assay to assess metabolic cell viability using Cell Counting Kit-8 (DOJINDO LABORATORIES, Kumamoto, Japan) [51,52]. Absorbance at 450 nm was measured using a microplate reader (iMark; Bio-Rad Laboratories, Inc., Hercules, CA, USA). Relative cell viability was calculated as a percentage of the absorbance of treated samples relative to that of controls. The following formula was used to obtain IC_50_ values, as previously reported [54]: IC_50_ = 10^[log(A/B) × (50−C)]/[(D−C) + Log(B)]^, where A and B are the corresponding concentrations of the test drug directly above and below 50% inhibition, respectively, and C and D are the percentages of inhibition directly below and above 50% inhibition, respectively. The propidium iodide (PI) uptake assay was performed to assess the percentage of dead cells as previously described [51,52]. Cells were incubated in situ with PI (1 μg/mL) and Hoechst 33342 (10 μg/mL) at 37 °C for 5 min in a CO_2_ incubator to stain dead cells and cell nuclei, respectively. The numbers of PI- and Hoechst-positive cells were scored under a fluorescence microscope (CKX53; OLYMPUS, Tokyo, Japan). The flow cytometry-based PI uptake assay was performed as described below. Cells were transfected with plasmids using Lipofectamine 2000, followed by a treatment with CTZ as described in the figure legends. To stain dead cells, cells were incubated with PI (1 μg/mL) at 37 °C for 5 min in a CO_2_ incubator, dissociated, and then washed with ice-cold PBS twice followed by fixation with 4% (*w*/*v*) paraformaldehyde at RT for 10 min. Flow cytometry was performed on FACSCanto II (BD Biosciences, Franklin Lakes, NJ, USA), and the data obtained were analyzed using FlowJo software, version 7.6.5 (Tree Star Inc., Ashland, OR, USA). A colony formation assay was conducted as previously described [10]. Cells were seeded at a low colony-forming density (200 cells/35 mm dish), cultured as described in the Figure legend, and then fixed with paraformaldehyde (4% *v*/*v*), followed by staining with crystal violet (0.1% *w*/*v*).

### 4.7. Data Reproducibility and Statistical Analysis

Cell viability/death assays, Western blotting, and RT-PCR analyses were repeated at least twice with similar results, and one set of representative data is presented. Data analyses were performed using the software Microsoft Excel (Version 2402 or Ver16.66.1, Redmond, Washington, WA, USA). Results are expressed as the mean and standard deviation (SD), and the significance of differences was assessed using Student’s two-tailed *t*-test for comparisons of two groups.

## Data Availability

All data are contained in this article and there are no repository data.
